# Epicardial VEGFC/D signaling is essential for coronary lymphangiogenesis

**DOI:** 10.1038/s44319-025-00431-7

**Published:** 2025-03-24

**Authors:** Ester de la Cruz, Vanessa Cadenas, Susana Temiño, Guillermo Oliver, Miguel Torres

**Affiliations:** 1 Cardiovascular Regeneration Program, Centro Nacional de Investigaciones Cardiovasculares (CNIC), Madrid, 28029 Spain; 2 Centro de Investigación Biomédica en Red de Enfermedades Cardiovasculares (CIBERCV), Madrid, Spain; 3 Center for Vascular and Developmental Biology, Feinberg Cardiovascular and Renal Research Institute, Northwestern University, Chicago, IL 60611 USA

**Keywords:** Vascular Biology, Lymphatic Vasculature, Heart Development, Coronary Vasculature, Sex-specific Gene Function, Development, Vascular Biology & Angiogenesis

## Abstract

The contractile ability of the mammalian heart critically relies on blood coronary circulation, essential to provide oxygen and nutrients to myocardial cells. In addition, the lymphatic vasculature is essential for the myocardial immune response, extracellular fluid homeostasis and response to injury. Recent studies identified different origins of coronary lymphatic endothelial cells, however, the cues that govern coronary lymphangiogenesis remain unknown. Here we show that the coronary lymphatic vasculature develops in intimate contact with the epicardium and with epicardial-derived cells. The epicardium expresses the lymphangiogenic cytokine VEGFC and its conditional deletion in the epicardium abrogates coronary lymphatic vasculature development. Interestingly, VEGFD is also expressed in the epicardium and cooperates with VEGFC in coronary lymphangiogenesis, but it does so only in females, uncovering an unsuspected sex-specific role for this cytokine. These results identify the epicardium/subepicardium as a signaling niche required for coronary lymphangiogenesis and VEGFC/D as essential mediators of this role.

## Synopsis

Signals from the epicardium are essential for fetal coronary lymphangiogenesis. VEGFC and VEGFD produced by the epicardium target the subepicardial space, where they are essential for the formation of the lymphatic vasculature in a dose-dependent manner.

- Epicardial *vegfc* deletion abrogates coronary lymphatic development.
- Epicardial *vegfc* overexpression expands the coronary lymphatic vasculature.
- VEGFD cooperates in coronary lymphangiogenesis with VEGFC in females but not in males, uncovering a new sex-specific role for VEGFD.
- Differences in the lymphangiogenic environment of the dorsal and ventral epicardium may underlie the different sensitivity to VEGFC/D deletion.

## Abstract (web summary)

Signals from the epicardium are essential for fetal coronary lymphangiogenesis. VEGFC and VEGFD produced by the epicardium target the subepicardial space, where they are essential for the formation of the lymphatic vasculature in a dose-dependent manner.

## Graphical abstract

**Figure:**
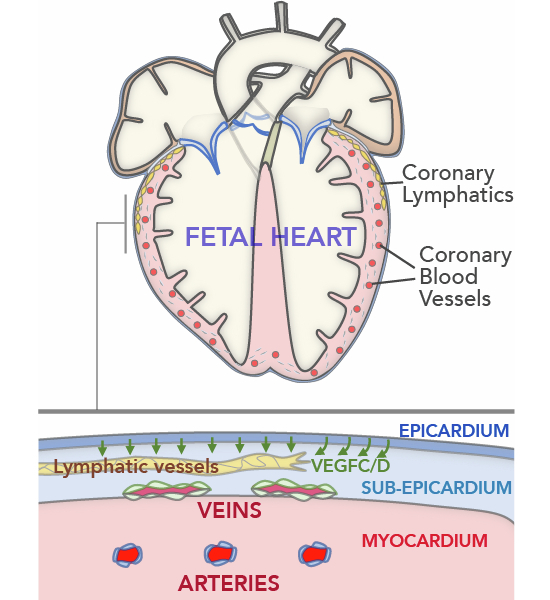

**Figure:**
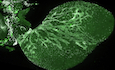

## Introduction

Besides the blood coronary vasculature, the ventricular myocardium also contains a fully developed network of cardiac lymphatics (Brakenhielm and Alitalo, 2019). Coronary lymphatics promote cardiomyocyte proliferation (Liu et al, 2020) and are important in the response to myocardial infarction (Henri et al, 2016; Klotz et al, 2015; Liu et al, 2020; Maruyama et al, 2021; Vieira et al, 2018) congestive heart failure (Witte et al, 1969), atherosclerosis (Lim et al, 2013; Milasan et al, 2016) and heart regeneration in zebrafish (Gancz et al, 2019; Harrison et al, 2019; Vivien et al, 2019). Cardiac lymphatics are first seen along the great arteries and sinus venosus at E12.5-E13.5 (Flaht et al, 2012; Karunamuni et al, 2010). Most embryonic and cardiac lymphatic endothelial cells (LECs) have a venous origin (Klotz et al, 2015; Wigle and Oliver, 1999); however, mesenchymal precursors from the second heart field (SHF) also contribute to ventral coronary LECs (Lioux et al, 2020; Maruyama et al, 2019). The transcription factor *Prox1* (Wigle and Oliver, 1999), as well as the growth factors VEGFC and VEGFD and their receptor VEGFR3 (Baldwin et al, 2005; Bower et al, 2017; Haiko et al, 2008; Karkkainen et al, 2004; Karkkainen et al, 2001; Paquet-Fifield et al, 2013) are essential during developmental lymphangiogenesis. In the heart, local signaling pathways like Retinoic acid (RA) and macrophage-derived hyaluronan promote cardiac lymphatic vessel maturation and remodeling (Cahill et al, 2021; Lioux et al, 2020), while the Sema3E-PlexinD1 axis is involved in coronary artery and lymphatic vessel patterning (Maruyama et al, 2021). Despite this progress, the cellular and molecular drivers of coronary lymphangiogenesis in the developing heart remain unknown. In some tissues/organs, arteries guide collector lymphatic vessel growth through VEGFC and CXCL12 expression from endothelial and smooth muscle cells (Cha et al, 2012; Vaahtomeri et al, 2017). Here, we characterize the growth pattern of coronary lymphatic vessels and report that they do not follow arteries or veins as they colonize the ventricles but, instead, rely on intimate interaction with the epicardium and epicardial-derived cells. Functional genetic analyses demonstrate that VEGFC/D are essential for the lymphangiogenic function of the epicardium.

## Results and discussion

**Figure 1:**
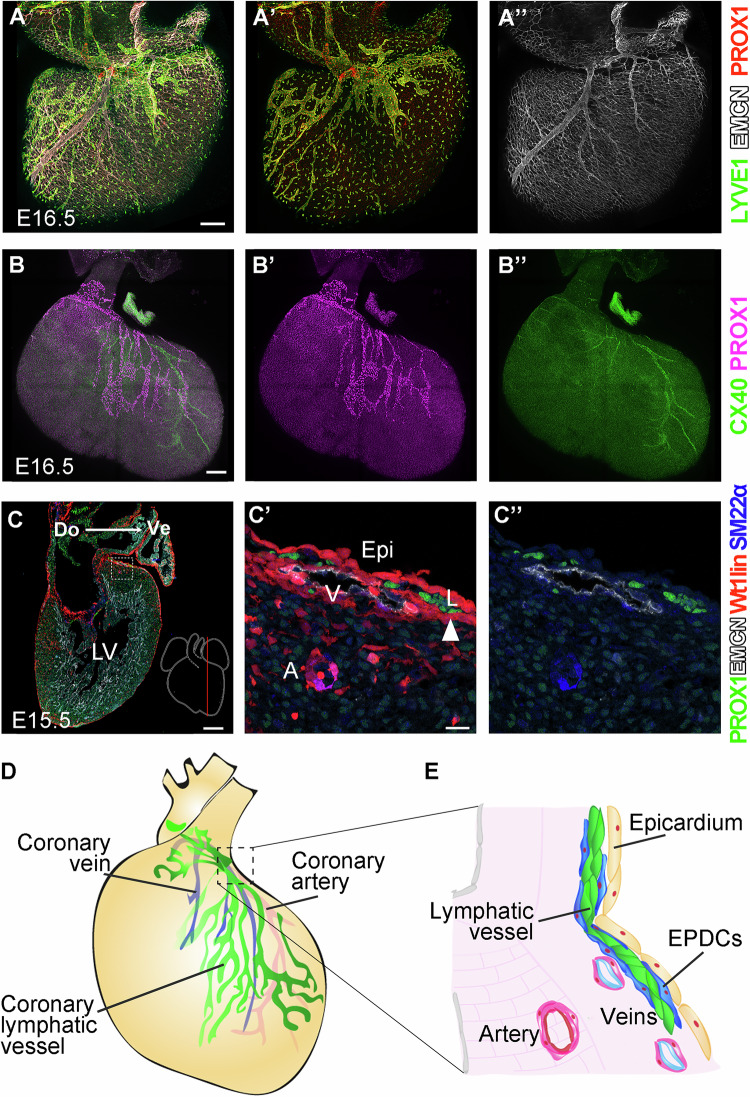
Subepicardial coronary lymphangiogenesis. **(A**–**A”**) Detection of veins with Endomucin (EMCN) and lymphatic vessels with Lyve1 and Prox1 in the dorsal side of E16.5 mouse hearts. (**B**–**B**”). Detection of arteries with Connexin 40 (CX40) and lymphatic vessels with PROX1 in the ventral side of an E16.5 mouse heart. (**C**–**C**”) Detection of arteries (A), veins (V), and lymphatic vessels (L) in a sagittal section of an E15.5 *Wt1*^*Cre*^*; Rosa26R*^*Tomato*^ mouse heart, which reveals the lineage of cells that express *Wt1*^*Cre*^ (Wt1lin), including the epicardium (Epi). Arrowhead in (**C**’) indicates lymphatic vessel-associated EPDCs. Do: dorsal; Ve: ventral; LV, left ventricle. (**D**, **E**) Schemes of the disposition of coronary vessels in a developing mouse heart. EPDC: epicardial-derived cells. Scale bars 200 µm in (**A**), (**B**), (**C**) and 20 µm in (**C**’).

To identify the mechanisms that guide coronary lymphangiogenesis, we studied the distribution of cardiac lymphatics as they grow into the ventricles of the mouse fetal heart (Fig. 1A–C”). Cardiac lymphatics appear at the base of the ventricles, dorsally at the sinus venosus and ventrally on the great arteries around embryonic day 12.5 (E12.5), however, they do not start colonizing the ventricles until E14.5 (reviewed in (Klaourakis et al, 2021). At E16.5, coronary veins and arteries are already present; however, lymphatic vessels do not follow the pattern of coronary arteries or veins as they colonize the ventricles (Fig. 1A–B”,D). In histological sections at E15.5 we observed that coronary lymphatic vessels grow in direct association with the epicardium (Fig. 1C–C”). Coronary veins also grow within the subepicardial space, however, when lymphatic vessels coincide with veins in the subepicardium, the lymphatic vessel is always located closer to the epicardium, representing the most superficial coronary vasculature (Fig. 1C’,C”,E). We thus found that coronary lymphatic vessels do not follow arteries or veins, as it happens in some tissues/organs (Cha et al, 2012; Vaahtomeri et al, 2017), but rather grow freely beneath the epicardium, without following either veins, which grow beneath the lymphatic vasculature, or arteries, which grow within the myocardium. This behavior correlates with the strong expression of VEGFC and CXCL12 in the epicardium (Cavallero et al, 2015; Chen et al, 2014).

**Figure 2:**
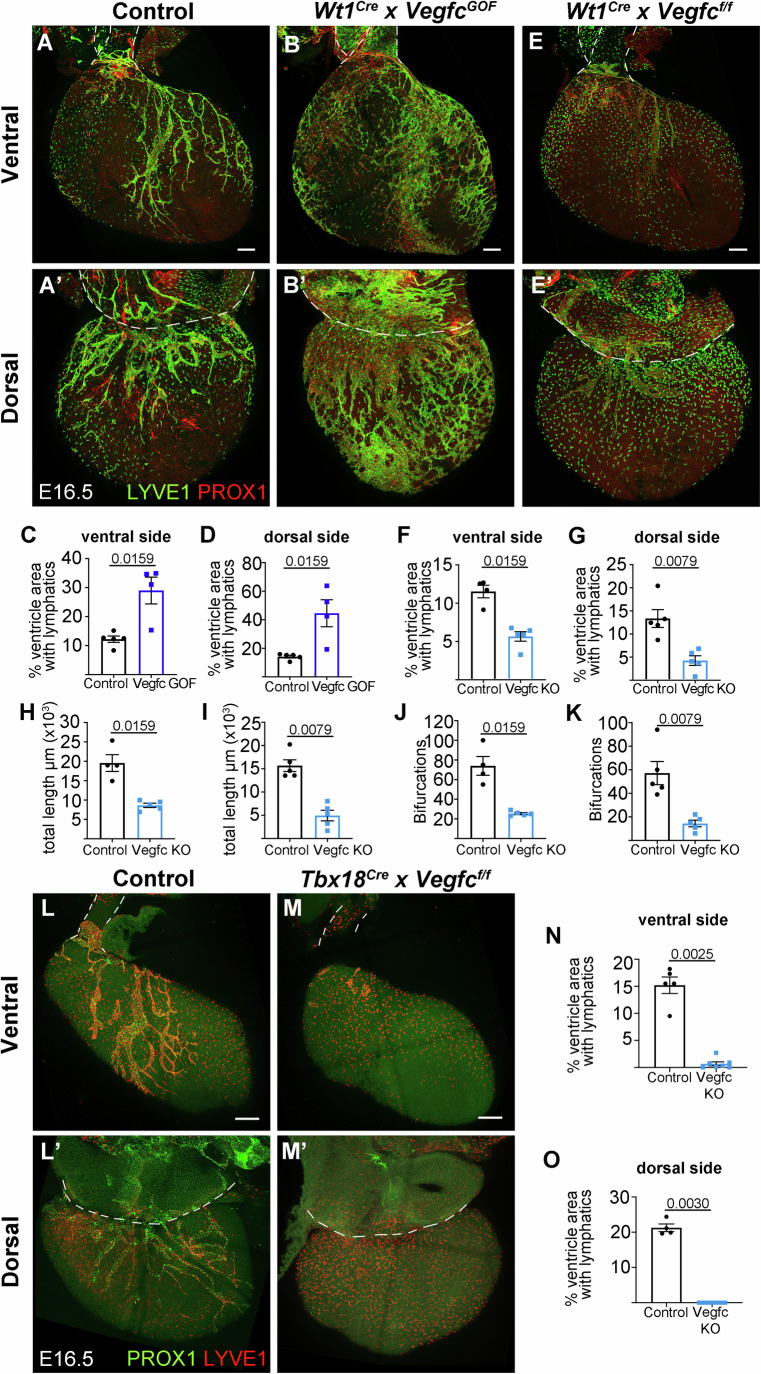
Epicardial VEGFC is essential for coronary lymphangiogenesis. (**A**–**D**) Representative specimens and quantifications of lymphatic vessel coverage of the dorsal and ventral ventricular surfaces upon *Vegfc* overexpression by activation of *Eef1a1*^*VegfcGOF*^ with *Wt1*^*Cre*^ (*Vegfc*^*GOF*^). (**E**–**K**) Representative specimens and quantifications of lymphatic vessel coverage, lymphatic length, and bifurcations of the dorsal and ventral ventricular surfaces upon *Vegfc* conditional elimination with *Wt1*^*Cre*^. (**L**–**O**) Representative specimens and quantifications of lymphatic vessel coverage of the dorsal and ventral ventricular surfaces upon *Vegfc* conditional elimination with *Tbx18*^*Cre*^. Statistics: Mann–Whitney test with two-tailed *p*-values. Graphs show mean ± standard deviation. Each dot represents one biological specimen. *N* = 5 control and 5 mutant hearts in each graph except in (**H**) and (**J**), in which 4 control hearts were analyzed, in (**N**), in which 7 mutant hearts were analyzed an in (**O**), in which 4 control and 7 mutant hearts were analyzed. Dotted lines in (**A**), (**B**), (**E**), (**L**), and (**M**) indicate the limits of the pulmonary artery (in front) and aorta (behind). Dotted lines in (**A**’), (**B**’), (**E**’), (**L**’), and (**M**’) indicate the limits of the sinus venosus. Scale bars 200 μm.

While VEGFC is strongly expressed in the epicardium (Chen et al, 2014), it is also expressed in other cardiac cells (Cahill et al, 2021); we therefore specifically studied the relevance of epicardial VEGFC in coronary lymphangiogenesis. We first studied the sensitivity of the developing lymphatic vasculature to increased epicardial VEGFC. For this, we used the *Eef1a1*^*VegfcGOF*^ allele (Pichol-Thievend et al, 2018), which provides conditional Cre-mediated VEGFC overexpression, combined with *Wt1*^*Cre*^ (Wessels et al, 2012), which drives Cre expression in the epicardium. Hearts with epicardial VEGFC overexpression showed increased coronary lymphatic coverage at E16.5 (Fig. 2A–D), indicating that coronary lymphangiogenesis is sensitive to epicardial VEGFC levels. Next, we studied the requirement for VEGFC function in the epicardium/EPDCs, by crossing *Vegfc*^*flox/flox*^ (Lim et al, 2019) with *Wt1*^*Cre*^ and analyzing the lymphatic vasculature at E16.5. Homozygous mutant hearts (*Vegfc*-KO) showed 50% reduction in lymphatic coverage in the ventral side of the ventricles and 68% reduction in the dorsal side (Fig. 2E–G). Total lymphatic length and lymphatic vessel bifurcations were also reduced in *Vegfc*-KO hearts (Fig. 2H–K). Epicardial *Vegfc*-KO mice were viable after birth and, as previously described (Liu et al, 2020), we observed a reduction in heart size at postnatal day 20 (P20) (Fig. EV1A).

**Figure EV1:**

Characterization of epicardial roles in Lymphangiogenesis. (**A**) Heart weight to body weight ratios (H/BW) in Control (*Vegf*^*f/f*^) and epicardial *Vegfc* KO (*WT1*^*Cre*^*;Vegf*^*f/f*^) hearts at postnatal day 20 (P20). (**B**) RNAseq quantification of Cre mRNA expression in dissected epicardial/subepicardial region from *Tbx18*^*Cre*^or *WT1*^*Cre*^ hearts. CPMs: counts per million. (**C**) RNAseq quantification of *Vegfc* mRNA exon 3 expression in dissected epicardial/subepicardial region from Control, *Tbx18*^*Cre*^; *Vegfc*^*f/f*^ or *WT1*^*Cre*^*; Vegfc*^*f/f*^ hearts (RPKM: Reads Per Kilobase Million). Statistics in (**A**), (**B**), according to the linear modeling in the LIMMA analysis software for RNAseq analysis (see Methods). Benjamini-Hochberg adjusted *P*-values are shown. Graphs show mean ± standard deviation. Each dot represents one biological specimen. *N* = 6 control and 5 *Vegfc*−/− hearts in (**A**) and (**B**). Statistics in (**C**): one-way ANOVA with Sidak’s correction for multiple comparisons. Graphs show mean ± standard deviation. Each dot represents one biological specimen. *N* = 8 control, 3 *WT1*^*Cre*^*;Vegf*^*f/f*^ and 5 *Tbx18*^*Cre*^; *Vegfc*^*f/f*^ hearts. (**D**) Detection of VEGFR3 expression by whole-mount immunofluorescence in wild type E16.5 Hearts. (**E**) Quantification of the mean intensity of VEGFR3 signal in coronary lymphatic vessels of the dorsal and ventral sides of the hearts shown in (**D**). (**F**) Detection of VEGFD expression by whole-mount immunofluorescence in wild type E16.5 Hearts. (**G**) Quantification of the mean intensity of VEGFD signal in coronary lymphatic vessels of the dorsal and ventral sides of the hearts shown in (**F**). (**H**) Detection of Cxcl12 expression by whole-mount immunofluorescence in wild type E16.5 Hearts. (**I**) Quantification of the mean intensity of Cxcl12 signal in coronary lymphatic vessels of the dorsal and ventral sides of the hearts shown in (**H**). Statistics in (**E**, **G**, **I**): Wilcoxon matched-pairs signed rank test with two-tailed *P*-values. Non-significant results are not indicated. Graphs show mean ± standard deviation. Each dot represents one biological specimen. *N* = 4 ventral sides and 5 dorsal sides in (**E**); 5 ventral and dorsal sides in (**G**) and 12 ventral and dorsal sides in (**I**). (**J**) Volcano plot representing the results of RNAseq comparing dorsal and ventral dissected epicardial/subepicardial regions from wild type E16.5 hearts. Genes encoding lymphangiogenic factors and differentially expressed appear highlighted in red. (**K**) Specific results from the RNAseq analysis shown in (**J**) for 22 selected genes encoding lymphangiogenic factors. Statistics in (**J**, **K**), according to the linear modeling in the LIMMA analysis software for RNAseq analysis (see Methods). Benjamini- Hochberg adjusted *P*-values are shown. When not shown, comparsions were not significant (*P*-value > 0.05). Scale bars 100 µm. Each dot represents a biological replicate. *N* = 3 ventral and 3 dorsal sides for all gene expressions represented.

To confirm these results with another epicardial Cre line, we induced epicardial deletion of *Vegfc* using *Tbx18*^*Cre*^ (Cai et al, 2008). Mutant hearts in this case lacked all lymphatic vessels of the dorsal side of the heart and showed only vestigial lymphatic vessels on the ventral side (Fig. 2L–O). These results suggest that the incomplete penetrance observed in the *Wt1*^*Cre*^-deleted hearts is due to lower efficacy of this Cre line compared with *Tbx18*^*Cre*^. Given that at mid-gestation, epicardial recombination mediated by *Tbx18*^*Cre*^ and *Wt1*^*Cre*^ is similar (Cai et al, 2008; Christoffels et al, 2009; Villa del Campo et al, 2016; Wessels et al, 2012), we studied the level of Cre expression in RNAseq of dissected epicardium/sub-epicardium of E16.5 mutant hearts recombined with either driver. We observed higher Cre expression in *Tbx18Cre* mice than in *Wt1Cre* mice (Fig. EV1B). To determine whether this affected the efficacy in *Vegfc*^*f*^ deletion, we performed similar RNAseq analysis in epicardial *Vegf*c mutants. We observed that elimination of *Vegfc* exon 3 was similarly efficient between Wt1^Cre^- and Tbx18^Cre^-recombined hearts at E16.5 (Fig. EV1C). This result suggests that timing of complete elimination of the VEGFC proteins underlies the different affection of the two genetic models. The fact that *Tbx18*^*Cre*^ already recombines the pro-epicardial organ (Cai et al, 2008; Tyser et al, 2021), whereas *Wt1*^*Cre*^ recombines extensively in the epicardium (Villa del Campo et al, 2016; Wessels et al, 2012) might also contribute to a different timing of VEGFC elimination between the two strains.

Dosed levels of epicardial VEGFC thus appear essential for coronary lymphangiogenesis, with more relevance in the dorsal than in the ventral side of the ventricles. We then studied VEGFR3 expression levels in the E16.5 dorsal and ventral coronary lymphatic vasculature and found no differences (Fig. EV1D,E). To then determine whether the difference between dorsal and ventral lymphangiogenesis could be determined by the signaling environment, we studied VEGFD and Cxcl12 expression by immunofluorescence (Fig. EV1F–I). Whereas we found no differences for VEGFD (Fig. EV1F,G), we observed about 25% higher expression of Cxcl12 on the dorsal side as compared to the ventral side of the heart (Fig. EV1H,I). To more broadly determine whether a different signaling environment might influence dorsal versus ventral coronary lymphangiogenesis, we then compared the transcriptomes of the dorsal and ventral epicardial/subepicardial regions of the E16.5 wild-type heart (Fig. EV1F) and determined the expression levels of 22 known extracellular regulators of lymphangiogenesis (Coso et al, 2014; Hussmann et al, 2023; Trincot et al, 2019; Zheng et al, 2014) (Fig. EV1G). This study showed that mRNAs coding for 8 of these factors were expressed in the ventral epicardium/sub-epicardium at higher levels than in the dorsal, whereas no one of them was expressed at higher levels dorsally (Fig. EV1J,K). This study suggests a generally more lymphangiogenic environment in the ventral subepicardium than in the dorsal one, however, some lymphangiogenic factors, like Cxcl12, appear more abundant dorsally at this stage. *Vegfc* is one of the factors with higher expression in the ventral epicardium, which suggests that the lower availability of this essential lymphangiogenic factor underlies the higher sensitivity of the dorsal lymphatic vasculature to *Vegfc* elimination.

**Figure 3:**
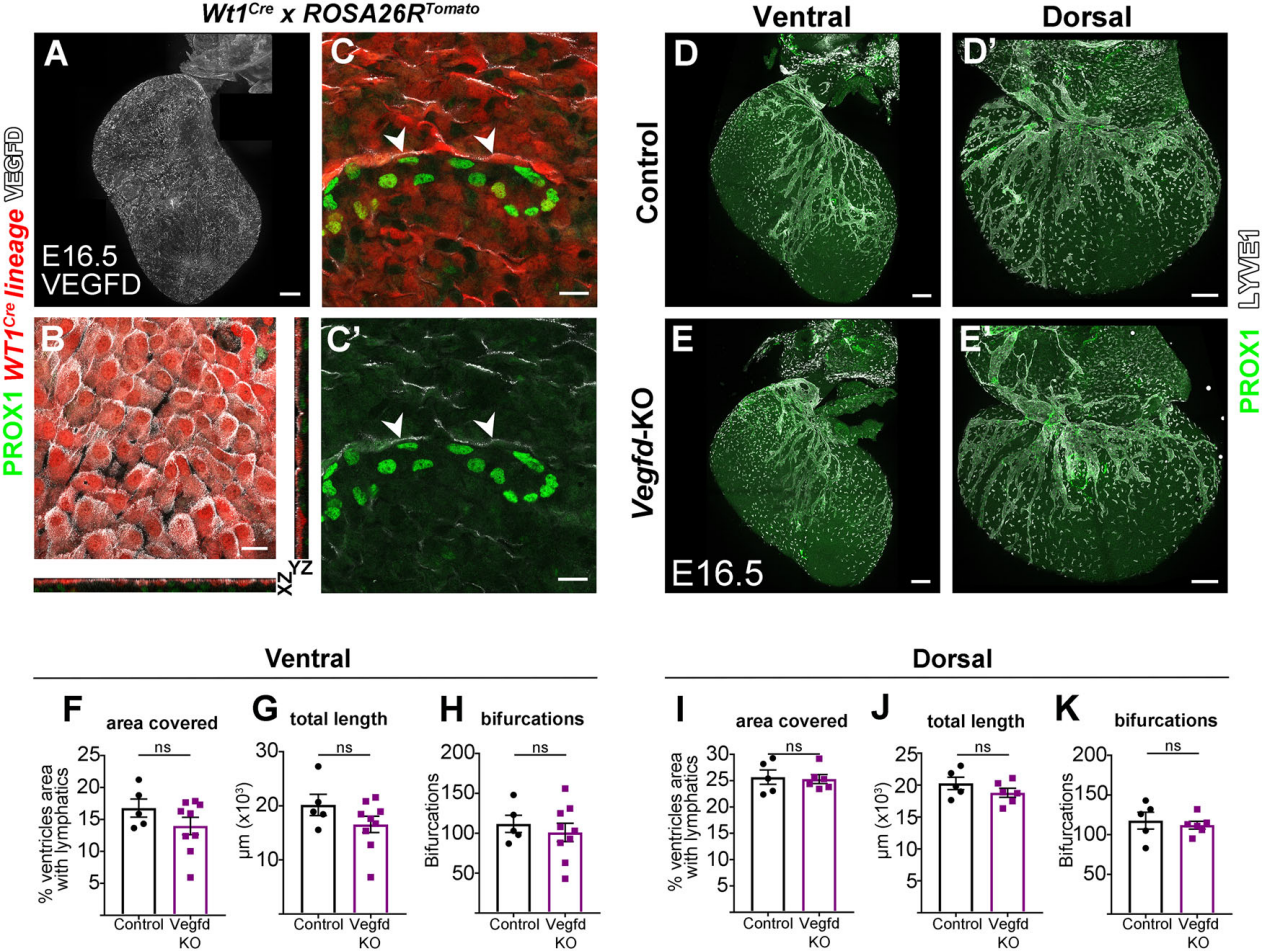
Epicardial expression of VEGFD is not essential for coronary lymphangiogenesis. (**A**) Confocal detection of VEGFD protein in a whole-mount E16.5 mouse heart (ventral side). This example corresponds to one of the specimens quantified and shown in Fig. EV2A. (**B**) Maximum projection of three epicardial confocal planes showing detection of VEGFD in the epicardium of a E16.5 *Wt1*^*Cre*^*; Rosa26R*^*Tomato*^ mouse heart, in which the *Wt1*^*Cre*^ lineage reveals the epicardium in red. (**C**–**C**’) Maximum projection of three subepicardial confocal planes showing Prox1^+^ lymphatic endothelial cells surrounded by VEGFD-expressing epicardial-derived cells (arrowheads). (**D**, **E**’) PROX1 and LYVE1 whole-mount immunostaining of E16.5 control and *Vegfd*^*−/−*^ (*Vegfd*-KO) hearts. (**F**–**K**) Quantification of the percentage of area covered by lymphatic vessels on the ventral and dorsal ventricular surfaces (**F**, **I**), total lymphatic length (**G**, **J**) and number of bifurcations (**H**, **K**) in control and *Vegfd*-KO hearts. Statistics: Mann–Whitney test with two-tailed *p*-values. Graphs show mean ± standard deviation. Each point represents a quantified heart. *N* = 5 control and 9 mutant hearts in (**F**–**G**) and 5 control and 6 mutant hearts in (**I**–**K**). Scale bars: 200 μm in (**A**, **D**, **E**’) and 20 μm in (**B**, **C**’).

**Figure EV2:**
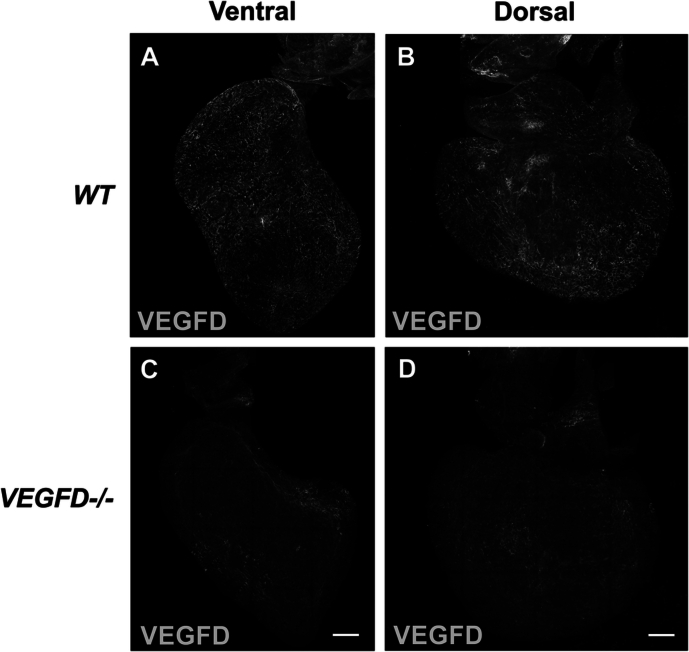
Characterization of VEGFD epicardial expression. (**A**, **B**) Detection of VEGFD expression by whole-mount immunofluorescence in wild type E16.5 hearts in ventral (**A**) and dorsal (**B**) views. The specimen in (**A**) is also shown as an example of the VEGFD expression pattern in Fig. 3A. (**C**, **D**) Equivalent detections in *Vegfd* knockout hearts. *N* = 5 Control and 2 VEGFD KO hearts. Scale bars 100 µm.

Although less prominent than VEGFC, VEGFD collaborates with VEGFC in promoting lymphangiogenesis in the developing intestinal lymphatic vasculature (Nurmi et al, 2015). Whole-mount detection of VEGFD protein in WT E16.5 hearts revealed expression in the epicardium (Fig. 3A,B) and epicardial-derived cells associated to the growing coronary lymphatic vasculature (Fig. 3C,C’). To functionally assess the role of VEGFD in coronary lymphatic development, we generated *Vegfd* global knockouts using CRISPR-Cas9 deletion of exons 3 and 4, which codify a portion of VEGFD central receptor-binding domain, “*VEGF homologous domain”* (VHD), essential for its function (Achen et al, 1998) (see Methods). Mice homozygous for this deletion showed a clear reduction of epicardial VEGFD detection by whole-mount immunofluorescence of E16.5 hearts (Fig EV2). As previously described, VEGFD deletion produced viable and fertile animals (Baldwin et al, 2005). The coronary lymphatic vasculature of *Vegfd* mutant hearts at E16.5 showed no significant alterations (Fig. 3D–K), although lymphatic vessel coverage, total lymphatic vessel length and total bifurcations showed a tendency to reduction on the ventral side of the ventricles (Fig. 3F–H).

**Figure 4:**
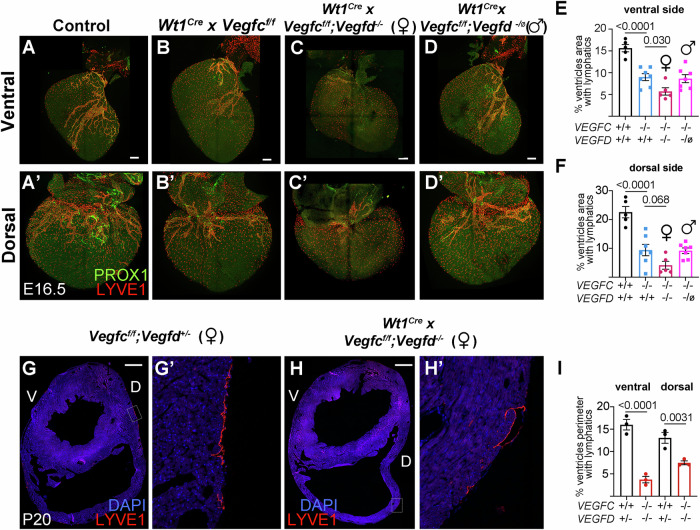
Sex-specific cooperative redundant roles of VEGFC and VEGFD in coronary lymphangiogenesis. (**A**–**D’**) Representative E16.5 specimens showing lymphatic vessel coverage of the dorsal and ventral ventricular surfaces upon combined conditional elimination of *Vegfc* with *Wt1*^*Cre*^ and constitutive elimination of *Vegfd*. (**E**, **F**) Quantification of the ventricular lymphatic vessel coverage in combinations of *Vegfc/d* mutants. Statistics: one-way ANOVA with Sidak’s correction for multiple comparisons. Graphs show mean ± standard deviation. Each dot represents one biological specimen. Scale bars: 100 μm. (**G**–**H’**) Representative sections of P20 specimens showing lymphatic vessel coverage of the dorsal and ventral ventricular surfaces upon combined conditional elimination of *Vegfc* with *Wt1*^*Cre*^ and constitutive elimination of *Vegfd*. (**G**’) and (**H**’) represent the boxed regions in (**G**) and (**H**), respectively. (**I**) Quantification of the ventricular lymphatic vessel coverage in combinations of *Vegfc/d* mutants at P20. Statistics: one-way ANOVA with Sidak’s correction for multiple comparisons. Graphs show mean ± standard deviation. Each dot represents one biological specimen. *N* = 5 wild type, 7 Vegfc−/−, 5 female *Vegfc/d* double KO and 7 male *Vegfc/d* double KO hearts in (**E**) and (**F**). *N* = 3 *Vegfd*+/− and 3 *Vegfc/d* double KO hearts in (**I**). Scale bars: 1 mm. D: dorsal; V: ventral. Significant *P*-values are shown on the graphs. When not shown, comparsions were not significant (*P*-value > 0.05).

**Figure EV3:**
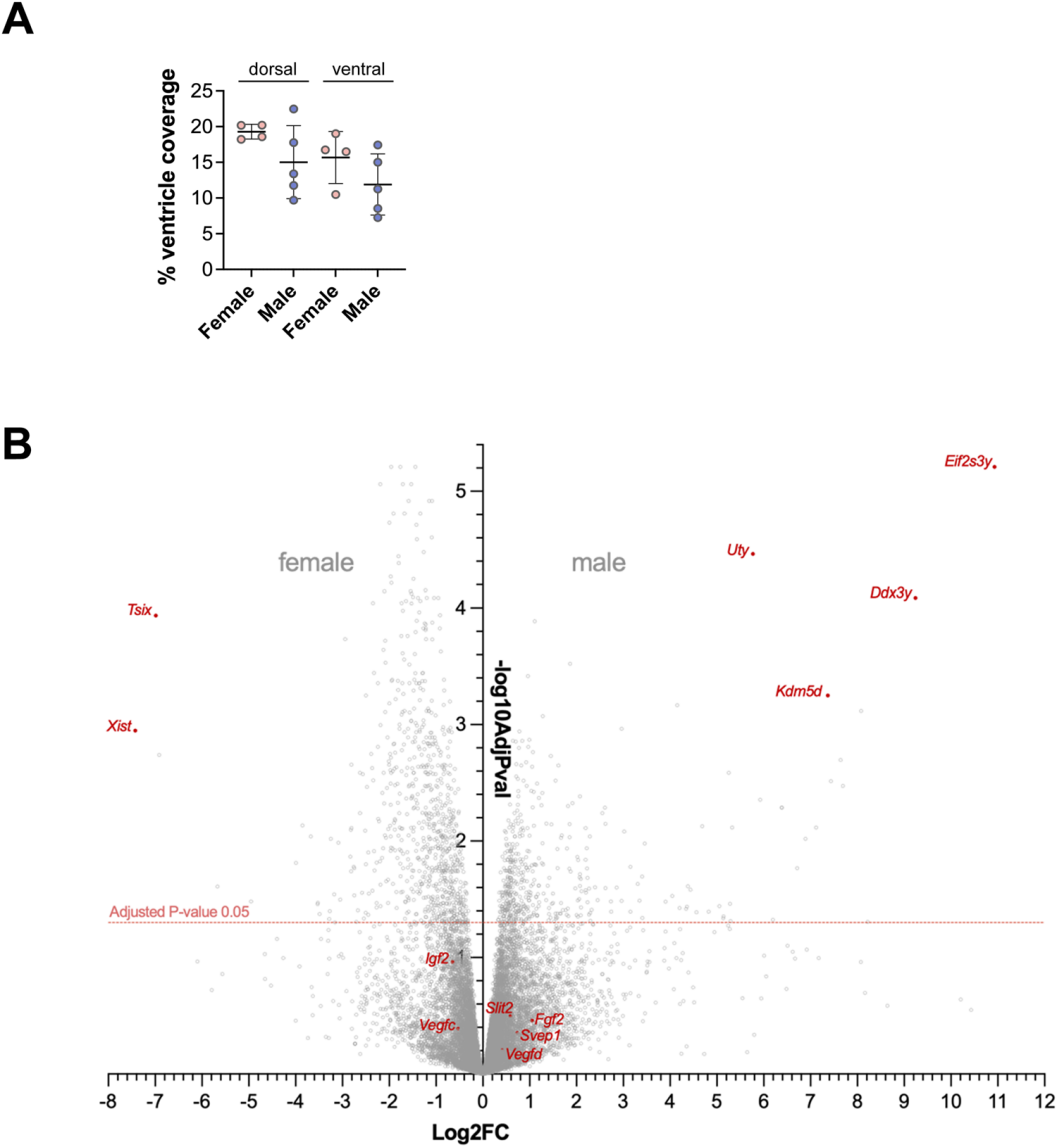
Analysis of sex-specific features of coronary lymphangiogenesis. (**A**) Quantification of lymphatic coverage on the ventricles of wild type E16.5 female and male hearts. Statistics: One-way ANOVA with Sidak’s multiple comparison test (non-significant). Graphs show mean ± standard deviation. Each dot represents one biological specimen. *N* = 4 female and 5 male samples for both the dorsal and the ventral sides. (**B**) Volcano plot representing the results of RNAseq comparing female to male dissected epicardial/subepicardial regions from wild type E16.5 hearts. Genes encoding for Y-linked and X-chromosome inactivation-specific genes appear in red. Some lymphangiogenic factors showing non-significant differential expression are highlighted in red. Statistics in (**B**), according to the linear modeling in the LIMMA analysis software for RNAseq analysis (see Methods). Benjamini-Hochberg adjusted *P*-values are shown.

To determine whether epicardial VEGFD and VEGFC act redundantly in cardiac lymphangiogenesis, we studied the coronary lymphatic vasculature of compound mutants (Fig. 4A–D’). We studied *Wt1*^*Cre*^-mediated epicardial elimination of *Vefgc* with or without elimination of *Vegfd*. Due to the location of *Vegfd* in the X chromosome, KO males are hemizygous, while KO females are homozygous for the *Vegfd* deletion. Elimination of *Vegfd* in females exacerbated the reduction in coronary lymphatic vasculature observed in the epicardial deletion of *Vegfc* (Fig. 4A–C’,E,F). This reduction was significant only in the ventral side but not on the dorsal side of the ventricles, suggesting a stronger interaction between VEGFC and VEGFD in the ventral side of the heart (Fig. 4E,F). Unexpectedly, we found that *Vegfd* deletion in males did not worsen the reduction in coronary lymphatics observed in the epicardial deletion of *Vegfc* (Fig. 4B,D,B’,D’,E,F). These results show that epicardial VEGFC and D cooperate in coronary lymphangiogenesis in females but not in males. Previous studies showed a higher lymphatic coverage in females than in males in adult hearts of the C57BL/6 mouse strain (Trincot et al, 2019). To determine whether this is also the case during gestation and in this genetic background, we quantified the lymphatic vessel coverage in control males and females of our VEGFC/VEGFD interaction study. We observed a non-significant tendency to a higher coverage in female hearts compared to male hearts (Fig. EV3A), on both the dorsal and the ventral sides. Sex-differences in coronary lymphatic coverage therefore may start to develop during gestation and are robust to changes in the genetic background. Together with the specific sensitivity of females to VEGFD loss, these observations suggest that enhanced coronary lymphangiogenesis might be driven by VEGFD signaling in females. To study the possible bases of the differential coronary lymphangiogenesis in males and females, we then studied the transcriptome of the epicardial/subepicardial region in E16.5 wild type female and male hearts. Whereas a large number of transcripts was found differentially expressed between males and females—including the expected male-specific Y-linked genes and female-specific X-inactivation genes—we did not observe differential expression for any of 22 known lymphangiogenic factors, including VEGFC and D (Fig. EV3B). These results suggest that the differences in coronary lymphatic vasculature between females and males are not dependent on differences in the sub-epicardial lymphangiogenic environment.

Finally, to determine whether the coronary lymphatic vasculature develops postnatally in the VEGFC/D mutant models generated, we studied epicardial VEGFC/D KO female hearts at P20. We found that the ventral coronary lymphatic vasculature suffers a 77% reduction at this stage, suggesting continuous requirement of epicardial VEGFC/D, whereas the dorsal vasculature shows a 41% reduction, suggesting partially postnatal recovery (Fig. 4G–I).

Our results thus reveal an essential role of the epicardium in coronary lymphangiogenesis during gestation and postnatally through the production of VEGFC and VEGFD. Previously, elimination of epicardial VEGFC showed delayed dorsal blood coronary vasculature development (Chen et al, 2014), however, here we show complete agenesis of the lymphatic vasculature, which reveals regulation of lymphangiogenesis by epicardial VEGFC and VEGFD independently of the blood vasculature. While we used epicardial drivers, epicardial-derived cells inherit this recombination, they associate intimately with LECs, and we showed that they express VEGFD. We cannot, therefore, exclude a role of epicardial-derived cells in contributing to coronary lymphangiogenesis. Nonetheless, the epicardium/subepicardium appears to be the most important driver of lymphangiogenesis, given that epicardial-derived cells invade both the sub-epicardium and the myocardium, whereas lymphatic vessels always grow in intimate contact with the epicardium, without entering the myocardium. This view is in agreement with the strong expression of the lymphatic endothelial cell guidance cytokine CXCL12 in the epicardium, together with VEGFC and VEGFD (Cavallero et al, 2015; Chen et al, 2014). Interestingly, the CXCL12/CXCR4 axis has recently been shown important for lymphangiogenesis and able to regulate the levels of VEGFR3 in LECS (Do et al, 2024). Another potential epicardial-derived cell population contributing to lymphangiogenesis through VEGFC/D production is the smooth muscle of the coronary vessels, however, single-cell transcriptomics has shown that smooth muscle cells express low levels of VEGFC compared to epicardium and fibroblasts (Feng et al, 2022). Furthermore, smooth muscle of the coronary vessels at the base of the ventricles mostly originates from neural crest and second heart field and therefore, their influence on lymphangiogenesis through VEGFC/D production would be intact in epicardial mutants; nonetheless, coronary lymphatics are fully eliminated from the base of the ventricles in *Tbx18*^*Cre*^ VEGFC mutants. These considerations, together with the lack of association of lymphatic vessels with great coronary blood vessels and their subepicardial location during pre-natal heart development strongly suggest that vascular smooth muscle does not play a significant role in the establishment of coronary lymphangiogenesis.

We also uncovered a sex-specific requirement for VEGFD in the heart, however, the role of VEGFD is only revealed in a defective VEGFC background, indicating that VEGFC alone is sufficient to drive coronary lymphangiogenesis in both males and females. Why males are insensitive to VEGFD deletion is difficult to characterize and may relate to sex-specific sensitivity to VEGFD signaling.

Finally, we report that the ventral subepicardium appears to be a more lymphangiogenic environment than the dorsal one, which may underlie differential affection of dorsal and ventral lymphatic vessels to mutations. The apparently different requirements of VEGFC and VEGFD in ventral versus dorsal lymphangiogenesis may also relate to the recent discovery of the contribution of second heart field precursors exclusively to the ventral coronary lymphatic endothelium (Lioux et al, 2020). The different origins of LECs in the ventral versus the dorsal side of the ventricles may determine intrinsic differential sensitivities of the ventral and dorsal lymphatic vasculatures to VEGFC and VEGFD, however, here we could not relate this differential sensitivity to differential expression of VEGFR3, so further studies will be required to elucidate this aspect.

In summary, we report a cardiac-specific mechanism of lymphatic vasculature development that relies on essential and redundant contributions of epicardial VEGFC and VEGFD to coronary lymphangiogenesis.

## Methods

**Table:** Reagents and tools table

Reagent/Resource	Reference or Source	Identifier or Catalog Number
**Experimental models**		
*Wt1*^*Cre*^ (*Mus musculus*)	Wessels et al, 2012	N/A
*Tbx18*^*Cre*^ (*Mus musculus*)	Cai et al, 2008	MGI:3801041
*Rosa26*^*tdtmt*^ (*Mus musculus*)	Madisen et al, 2010	MGI:3809523
*Vegfc*^*flox*^ (*Mus musculus*)	Lim et al, 2019	N/A
*Eef1a1*^*VegfcGOF*^(*Mus musculus*)	Pichol-Thievend et al, 2018	N/A
*Vegfd*^*–*^ (*Mus musculus*)	This study	Methods and Protocols
**Antibodies**		
CX-40	Alpha Diagnostics	CX40-A
EMCN eFluor660	eBioscience^TM^	50-5851-82
LYVE1	ReliaTech	03-PA50S
PROX1	R&D systems	AF2727
SM22α	Abcam	ab14106
VEGFD	Cusabio CSB-	CSB-PA07554A0Rb
VEGFR3	R&D Systems	AF743
CXCL12	R&D Systems	MAB350
Goat anti-Rabbit 405	Thermo Fisher	A31556
Goat anti-Rabbit 488	Life Technologies^TM^	A11034
Donkey anti-Rabbit 488	Molecular Probes	A21206
Goat anti-Rabbit 633	Life Technologies^TM^	A21071
Donkey anti-Rabbit 633	Sigma	SAB4600132
Donkey anti-Rabbit Cy5	Jackson Immuno	711-495-152
Goat anti-Rat 488	Thermo Fisher	A11006
Donkey anti-Rat 488	Molecular Probes	A21208
Donkey anti-Rat Cy5	Jackson Immuno	712-175-150
Donkey anti-Goat 488	Thermo Fisher	A11055
Donkey anti-Goat 647	Thermo Fisher	A21447
Goat anti-Mouse 633	Life Technologies^TM^	A21052
**Oligonucleotides and other sequence-based reagents**		
Fw flanking Vegfd exon 3	This study	Methods and Protocols
Rev flanking Vegfd exon 3	This study	Methods and Protocols
Fw flanking Vegfd exon 4	This study	Methods and Protocols
Rev flanking Vegfd exon 4	This study	Methods and Protocols
Fw flanking Vegfd exons 3 and 4	This study	Methods and Protocols
Rev flanking Vegfd exons 3 and 4	This study	Methods and Protocols
sgRNA targeted to Vegfd exon 2	This study	Methods and Protocols
sgRNA targeted to Vegfd exon 2	This study	Methods and Protocols
sgRNA targeted to Vegfd exon 4	This study	Methods and Protocols
sgRNA targeted to Vegfd exon 4	This study	Methods and Protocols
**Chemicals, Enzymes and other reagents**		
Evolve-KSOM medium	Zenith Biotech	ZEKS-050
S.p. HiFi Cas9 Nuclease V3	IDT Alt-R®	1081060
KCl	Sigma	P9541
Sucrose	Sigma	16104
Gelatin	Sigma	G2500
Isopentane	Sigma	1060561000
Triton X-100	Sigma	T9284
TNB blocking reagent	Perkin-Elmer	FP1012
Tween-20	Sigma	P9416
Fluorescence mounting medium	Dako	s3023
RNeasy Micro kit	Qiagen	74004
NEBNext Ultra RNA Library preparation kit	New England Biolabs	NEB #E7770
**Software**		
ImageJ	https://imagej.net/ij/	Version 1.54 g
bcl2fastq	Illumina	v2.20.0.422
FastQC	http://www.bioinformatics.babraham.ac.uk/projects/fastqc/	Version 0.12.0
Cutadapt	Martin, 2011	Version 1.7.1
RSEM	https://github.com/deweylab/RSEM	v1.2.20
Bioconductor package LIMMA	Ritchie et al, 2015	Version 3.20

### Mouse lines

Animals were handled in accordance with CNIC Ethics Committee, Spanish laws and the EU Directive 2010/63/EU for the use of animals in research. All mouse experiments were approved by the CNIC and Universidad Autónoma de Madrid Committees for “Ética y Bienestar Animal” and the area of “Protección Animal” of the Community of Madrid with references PROEX 220/15 and PROEX 144.1/21 For this study, mice were maintained on a mixed background. Mouse experiments performed at Northwestern University were performed in accordance with protocols approved by Northwestern University Institutional Animal Care and Use Committee. The mouse alleles used here and already described were: *Wt1*^*Cre*^ (Wessels et al, 2012), *Tbx18*^*Cre*^ (Cai et al, 2008), *Rosa26*^*tdtmt*^ (Madisen et al, 2010), *Vegfc*^*flox*^ (Lim et al, 2019), *Eef1a1*^*VegfcGOF*^ (Pichol-Thievend et al, 2018).

A new *Vegfd knockout* line was generated using CRISPR-Cas9 technology. Four sgRNAs that recognized *Vegfd* genomic sequences were designed using the CRISPOR web tool (Concordet and Haeussler, 2018): two sgRNAS to target intron 2 (A and B), and two to target intron 4 (C and D). sgRNA-coding sequences (A) TAGGTTAAGTTCCCATATAGTGG; (B) GCGTCATGAAAAGCATGTCAGGG; (C) ATGCCTGTATAATGGGTAAAGG; (D) GTGCAACACATGTCTTTCTG AGG. The four sgRNAs were used simultaneously. Around 3.4 kb of *Vegfd* gene from intron 2 to intron 4 and including exon 3, intron 3 and exon 4 were deleted. Exons 3–4 code for VEGFD amino acids 106 to 218. To generate mouse mutants carrying the deletion ten 3 to 5-weeks old C57BL/6JCrl females were superovulated by injection of 5 IU of PMSG and 48 h later with 5 IU of hCG. Females were then crossed with C57BL/6JCrl males and the next morning they were checked for positive plug. Fertilized zygotes were retrieved and incubated at 37 °C with 5% CO_2_/5% O_2_ in Evolve-KSOM medium (Zenith Biotech ZEKS-050) for pronuclear microinjection of 1–2 pL containing 100 μg of Cas9 protein (IDT Alt-R® S.p. HiFi Cas9 Nuclease V3, 1081060) and 0.305 μM of each sgRNA (A to D) (IDT Alt-R® CRISPR-Cas9 sgRNA). Injected embryos were then incubated in Evolve-KSOM medium at 37 °C and 5% CO_2_/5% O_2_ overnight. The next morning, the embryos at two-cell stage were transferred to CD-1 pseudopregnant females. 5 microinjected animals were obtained at weaning and 3 of them had exons 3 and 4 deleted and were used to establish the mouse line. For genotyping, the following primers were used: To detect the deletion of Exon 3: Fw1: GTGCTATCCAGCTGTAGCCT; Rv1: CCCCTGAGCCTGTTTCTTTACT; To detect the deletion of Exon 4: Fw2: GGGCAAAAATGCAGATGGTGG; Rv2: GATCCTCAAGGTTTTGGGTCCT; To detect the total deletion of both, exon 3 and exon 4: Fw1: GTGCTATCCAGCTGTAGCCT; Rv2: GATCCTCAAGGTTTTGGGTCCT. The specific deletions obtained were characterized by Sanger DNA sequencing. We confirmed that the mutant sequences aligned with the flanking regions of *Vegfd* exons 3 and 4 and contained deletions from 276 bp upstream exon 3 to 67 bp downstream exon 4 (2 founders) or from 272 bp upstream exon 3 to 90 bp downstream exon 4 (1 founder). Females were maintained in heterozygosity and the males in hemizygosity. These animals were crossed and maintained in a C57BL/6 background. Mouse strains described here are available upon request.

Except for *Vegfd*-KO line, mice were genotyped by PCR as described in the original reports. Male and female mice older than 8 weeks of age were used for mating. Experimental specimens were retrieved during gestation and sex-determined by PCR for *Vegfd*-KO and *Vegfd*-KO;*Vegfc*-KO embryos.

### Embryo and organ retrieval

The morning in which the vaginal plug was detected was considered as embryonic day 0.5 (E0.5). Pregnant females were sacrificed by CO_2_ inhalation followed by cervical dislocation. Embryos were dissected in PBS with a sprinkle of Heparin (ROVI 1000 IU/mL). Fetuses were decapitated and placed in a 50 mM KCl (Sigma P9541) solution in PBS with heparin to stop the hearts in diastole and avoid clotting before dissection. Hearts were dissected and fixed in PFA 4% in PBS overnight at 2 °C. The tip of the tail was used to genotype specimens.

### Tissue sectioning and immunostaining

Hearts were washed in PBS after fixation and cryoprotected in 15% sucrose (Sigma, 16104) PBS overnight at 4 °C. The next morning, sucrose was removed and a 37 °C pre-heated solution of 7.5% gelatin (Sigma, G2500), 15% sucrose in PBS was added. Hearts were incubated in this solution for at least 4 h and then allowed to solidify at 4 °C overnight. Gelatin blocks were snap-frozen in a −70 °C solution of isopentane (Sigma, 1060561000) for 1 min. The frozen blocks were kept at −80 °C until sectioned. 8-μm-thick cryo-sections were made using a Leica CM1950 automated Cryostat and stored at −20 °C until used. Slides were thawed at room temperature and gelatin was removed from the slides by two 10-min washes with PBS at 37 °C and a quick wash with room-temperature (RT) PBS. Sections were then permeabilised with 0.5% Triton X-100 (Sigma T9284) in PBS for 30 min at RT, followed by PBS washing and treated with TNB blocking reagent (Perkin Elmer, FP1012) for 1 h at RT. The sections were then incubated with primary-antibody dilutions prepared in the same TNB blocking solution within a humid chamber at 4 °C overnight. Secondary antibodies were incubated for 1 h at RT. Slides were washed with 0.01% Tween-20 (Sigma P9416) in PBS several times between the previous steps. Slides were mounted with Dako fluorescence mounting medium (s3023).

### Antibodies

CX-40: Alpha Diagnostics CX40-A, Rabbit 1:200; EMCN eFluor660 eBioscience^TM^ 50-5851-82, Rat 1:100/1:200; LYVE1 ReliaTech 03-PA50S, Rabbit 1:200; PROX1 R&D systems AF2727, Goat 1:200; SM22α abcam ab14106, Rabbit 1:200; VEGFD Cusabio CSB-PA07554A0Rb, Rabbit 1:200; VEGFR3 R&D Systems AF743, Goat 1:100/1:200; CXCL12 R&D Systems MAB350, Mouse 1:100.

### Whole-mount immunofluorescence

For hearts, after fixation and subsequent wash with PBS, all the following steps were performed at 4 °C in a rotating wheel. 500 μL were added at each step, except for the washes that were performed in a 50 ml Falcon tube. Protocol: Permeabilization for 2 days in 0.5% Triton X-100 in PBS was followed by 2-h wash with PBS. TNB blocking was performed over day and primary antibody incubation for 3 days followed by several washes over day with 0.01% Tween 20 in PBS. Secondary antibodies were incubated from overnight to 2 days, depending on the antigen and cardiac stage. Finally, hearts were thoroughly washed with 0.01% Tween 20 in PBS followed by PBS to remove detergent. To improve image acquisition while preserving fluorescence, hearts were mildly clarified with increasing glycerol concentrations starting at 20% glycerol in PBS up to 80%. For immunofluorescences of Fig. 2, the blocking solution was 3% BSA, 0.1% Triton, and 5% donkey serum in PBS.

### Image acquisition and quantification

Whole-mount and cardiac sections immunofluorescence were acquired with a Nikon W1 Spinning Disk inverted confocal microscope for Fig. 2A–E’. For the rest of the images, a Leica TCS SP8 coupled to a DMi8 inverted microscope was used with Navigator module and equipped with a white light laser. Images were analyzed and quantifications were made using ImageJ (http://rsb.info.nih.gov/ij). Maximum projections were acquired using TileScan and z-stack functions. The Maximum Projection of equivalent z-stacks for the different hearts was used for quantification. We used the Freehand selection tool of ImageJ to select the whole ventral or dorsal ventricular surfaces and the area covered by Lymphatic vessels. For LYVE1 whole-mount quantifications of Vegfd-KOs, Vegfc-GOF and VEGFC-KO;VEGFD-KO hearts, a macro was used to quantify lymphatic coverage. This included several steps: duplicate channel of interest, median filter, binary conversion to Mask, analyze particles tool to remove smaller particles (macrophages) and finally, after creating a raw selection, manual curation using the Freehand selection tool. To quantify the total lymphatic length the Freehand line tool was used to draw lines running medial along each lymphatic branch. Total length was obtained by adding the length of every branch. Bifurcations were manually counted, considering each intersection point of two or more lymphatic branches.

### RNAseq analysis

The epicardium/subepicardium was dissected as previously described (Lioux et al, 2020) and quickly frozen in separate 1.5 ml Eppendorf tubes on dry ice. For mRNA isolation, samples from at least three different litters were used. As low RNA amount from one single epicardial layer was expected, two dissected epicardia of the same genotype were pooled together. Therefore, the three biological replicates per genotype represent six different hearts pooled by pairs. RNA was then purified from each pool using RNeasy Micro kit (Qiagen 74004). 40 ng of total RNA were used to generate barcoded RNA-seq libraries using the NEBNext Ultra RNA Library preparation kit (New England Biolabs). Briefly, poly A + RNA was purified using poly-T oligo-attached magnetic beads followed by fragmentation and then first and second cDNA strand synthesis. Next, cDNA 3’ ends were adenylated and the adapters were ligated followed by PCR library amplification. Finally, the size of the libraries was checked using the Agilent 2100 Bioanalyzer DNA 1000 chip and their concentration was determined using the Qubit® fluorometer (Life Technologies). Libraries were sequenced on a HiSeq2500 (Illumina) to generate 60 bases single reads and processed with RTA v1.18.66.3. FastQ files for each sample were obtained using bcl2fastq v2.20.0.422 software (Illumina). The raw data analysis of RNA-seq data were generated by CNIC Bioinformatics unit. Read quality was assessed with FastQC (http://www.bioinformatics.babraham.ac.uk/projects/fastqc/). Illumina adapter sequences were trimmed with Cutadapt 1.7.1 (Martin, 2011), which also discarded reads that were shorter than 30 bp. The resulting reads were mapped against the mouse transcriptome (GRCm38, release 91; aug2017 archive) and quantified using RSEM v1.2.20. Data were then processed with a differential expression analysis pipeline that used Bioconductor package LIMMA (Ritchie et al, 2015) for normalization and differential expression testing. Genes with at least 1 count per million in at least 3 samples were considered for statistical analysis. We considered as differentially expressed those genes with Benjamin-Hochberg adjusted *p*-value < 0.05. Fold change and log (ratio) values were calculated to represent gene expression differences between conditions. For pathways analysis, the Ingenuity Pathway Analysis software was used.

### Statistics

The details of each test used are specified in Figure legends. As a general rule, Mann–Whitney two-tailed test was performed to compare two groups of quantitative data. One-way ANOVA was performed to compare more than two groups of quantitative data with one independent variable, after a normality test (Kolmogorov–Smirnov) was found positive for all samples. Data is indicated as mean ± SEM of the individual plotted values. All comparisons and graphs were made using GraphPad Prism 9 statistical analysis software. In all cases *P*-values were two-tailed and adjusted by Sidak’s correction for multiple measurements. Values of *p* ≥ 0.05 were considered non-significant. No blinding was used in the experiments.

## Supplementary information

Peer Review File

Expanded View Figures

## Data Availability

Sequencing data have been deposited to GEO with accession number GSE284800. Source data are available at Mendeley (https://data.mendeley.com/datasets/4dhff9k2xb/2). The source data of this paper are collected in the following database record: biostudies:S-SCDT-10_1038-S44319-025-00431-7.

## References

[CR1] Achen MG, Jeltsch M, Kukk E, Mkinen T, Vitali A, Wilks AF, Alitalo K, Stacker SA (1998) Vascular endothelial growth factor D (VEGF-D) is a ligand for the tyrosine kinases VEGF receptor 2 (Flk1) and VEGF receptor 3 (Flt4). Proc Natl Acad Sci USA 95:548–553 9435229 10.1073/pnas.95.2.548 PMC18457

[CR2] Baldwin ME, Halford MM, Roufail S, Williams RA, Hibbs ML, Grail D, Kubo H, Stacker SA, Achen MG (2005) Vascular endothelial growth factor D is dispensable for development of the lymphatic system. Mol Cell Biol 25:2441–2449 15743836 10.1128/MCB.25.6.2441-2449.2005 PMC1061605

[CR3] Bower NI, Vogrin AJ, Le Guen L, Chen H, Stacker SA, Achen MG, Hogan BM (2017) Vegfd modulates both angiogenesis and lymphangiogenesis during zebrafish embryonic development. Development 144:507–518 28087639 10.1242/dev.146969

[CR4] Brakenhielm E, Alitalo K (2019) Cardiac lymphatics in health and disease. Nat Rev Cardiol 16:56–68 30333526 10.1038/s41569-018-0087-8

[CR5] Cahill TJ, Sun X, Ravaud C, Villa Del Campo C, Klaourakis K, Lupu IE, Lord AM, Browne C, Jacobsen SEW, Greaves DR et al (2021) Tissue-resident macrophages regulate lymphatic vessel growth and patterning in the developing heart. Development 148:dev194563 33462113 10.1242/dev.194563 PMC7875498

[CR6] Cai CL, Martin JC, Sun Y, Cui L, Wang L, Ouyang K, Yang L, Bu L, Liang X, Zhang X et al (2008) A myocardial lineage derives from Tbx18 epicardial cells. Nature 454:104–108 18480752 10.1038/nature06969 PMC5540369

[CR7] Cavallero S, Shen H, Yi C, Lien CL, Kumar SR, Sucov HM (2015) CXCL12 signaling is essential for maturation of the ventricular coronary endothelial plexus and establishment of functional coronary circulation. Dev Cell 33:469–477 26017771 10.1016/j.devcel.2015.03.018 PMC4448078

[CR8] Cha YR, Fujita M, Butler M, Isogai S, Kochhan E, Siekmann AF, Weinstein BM (2012) Chemokine signaling directs trunk lymphatic network formation along the preexisting blood vasculature. Dev cell 22:824–836 22516200 10.1016/j.devcel.2012.01.011 PMC4182014

[CR9] Chen HI, Sharma B, Akerberg BN, Numi HJ, Kivela R, Saharinen P, Aghajanian H, McKay AS, Bogard PE, Chang AH et al (2014) The sinus venosus contributes to coronary vasculature through VEGFC-stimulated angiogenesis. Development 141:4500–4512 25377552 10.1242/dev.113639 PMC4302936

[CR10] Christoffels VM, Grieskamp T, Norden J, Mommersteeg MT, Rudat C, Kispert A (2009) Tbx18 and the fate of epicardial progenitors. Nature 458:E8–9 19369973 10.1038/nature07916

[CR11] Concordet JP, Haeussler M (2018) CRISPOR: intuitive guide selection for CRISPR/Cas9 genome editing experiments and screens. Nucleic Acids Res 46:W242–W245 29762716 10.1093/nar/gky354 PMC6030908

[CR12] Coso S, Bovay E, Petrova TV (2014) Pressing the right buttons: signaling in lymphangiogenesis. Blood 123:2614–2624 24608974 10.1182/blood-2013-12-297317

[CR13] Do LNH, Delgado E, Lim CG, Bkhache M, Peluzzo AM, Hua Y, Oza M, Mohsin S, Chen H, Autieri MV et al (2024) A neuro-lymphatic communication guides lymphatic development by CXCL12 and CXCR4 signaling. Development 151:dev202901 39470100 10.1242/dev.202901 PMC11634036

[CR14] Feng W, Bais A, He H, Rios C, Jiang S, Xu J, Chang C, Kostka D, Li G (2022) Single-cell transcriptomic analysis identifies murine heart molecular features at embryonic and neonatal stages. Nat Commun 13:7960 36575170 10.1038/s41467-022-35691-7 PMC9794824

[CR15] Flaht A, Jankowska-Steifer E, Radomska DM, Madej M, Gula G, Kujawa M, Ratajska A (2012) Cellular phenotypes and spatio-temporal patterns of lymphatic vessel development in embryonic mouse hearts. Dev Dyn 241:1473–1486 22753134 10.1002/dvdy.23827

[CR16] Gancz D, Raftrey BC, Perlmoter G, Marin-Juez R, Semo J, Matsuoka RL, Karra R, Raviv H, Moshe N, Addadi Y et al (2019) Distinct origins and molecular mechanisms contribute to lymphatic formation during cardiac growth and regeneration. eLife 8:e44153 31702554 10.7554/eLife.44153 PMC6881115

[CR17] Haiko P, Makinen T, Keskitalo S, Taipale J, Karkkainen MJ, Baldwin ME, Stacker SA, Achen MG, Alitalo K (2008) Deletion of vascular endothelial growth factor C (VEGF-C) and VEGF-D is not equivalent to VEGF receptor 3 deletion in mouse embryos. Mol Cell Biol 28:4843–4850 18519586 10.1128/MCB.02214-07 PMC2493372

[CR18] Harrison MR, Feng X, Mo G, Aguayo A, Villafuerte J, Yoshida T, Pearson CA, Schulte-Merker S, Lien CL (2019) Late developing cardiac lymphatic vasculature supports adult zebrafish heart function and regeneration. eLife 8:e42762 31702553 10.7554/eLife.42762 PMC6881116

[CR19] Henri O, Pouehe C, Houssari M, Galas L, Nicol L, Edwards-Levy F, Henry JP, Dumesnil A, Boukhalfa I, Banquet S et al (2016) Selective stimulation of cardiac lymphangiogenesis reduces myocardial edema and fibrosis leading to improved cardiac function following myocardial infarction. Circulation 133:1484–1497 26933083 10.1161/CIRCULATIONAHA.115.020143

[CR20] Hussmann M, Schulte D, Weischer S, Carlantoni C, Nakajima H, Mochizuki N, Stainier DYR, Zobel T, Koch M, Schulte-Merker S (2023) Svep1 is a binding ligand of Tie1 and affects specific aspects of facial lymphatic development in a Vegfc-independent manner. eLife 12:e82969 37097004 10.7554/eLife.82969 PMC10129328

[CR21] Karkkainen MJ, Haiko P, Sainio K, Partanen J, Taipale J, Petrova TV, Jeltsch M, Jackson DG, Talikka M, Rauvala H et al (2004) Vascular endothelial growth factor C is required for sprouting of the first lymphatic vessels from embryonic veins. Nat Immunol 5:74–80 14634646 10.1038/ni1013

[CR22] Karkkainen MJ, Saaristo A, Jussila L, Karila KA, Lawrence EC, Pajusola K, Bueler H, Eichmann A, Kauppinen R, Kettunen MI et al (2001) A model for gene therapy of human hereditary lymphedema. Proc Natl Acad Sci USA 98:12677–12682 11592985 10.1073/pnas.221449198 PMC60113

[CR23] Karunamuni G, Yang K, Doughman YQ, Wikenheiser J, Bader D, Barnett J, Austin A, Parsons-Wingerter P, Watanabe M (2010) Expression of lymphatic markers during avian and mouse cardiogenesis. Anat Rec 293:259–270 10.1002/ar.21043 PMC3607319 19938109

[CR24] Klaourakis K, Vieira JM, Riley PR (2021) The evolving cardiac lymphatic vasculature in development, repair and regeneration. Nat Rev Cardiol 18:368–379 33462421 10.1038/s41569-020-00489-x PMC7812989

[CR25] Klotz L, Norman S, Vieira JM, Masters M, Rohling M, Dube KN, Bollini S, Matsuzaki F, Carr CA, Riley PR (2015) Cardiac lymphatics are heterogeneous in origin and respond to injury. Nature 522:62–67 25992544 10.1038/nature14483 PMC4458138

[CR26] Lim HY, Thiam CH, Yeo KP, Bisoendial R, Hii CS, McGrath KC, Tan KW, Heather A, Alexander JS, Angeli V (2013) Lymphatic vessels are essential for the removal of cholesterol from peripheral tissues by SR-BI-mediated transport of HDL. Cell Metab 17:671–684 23663736 10.1016/j.cmet.2013.04.002

[CR27] Lim L, Bui H, Farrelly O, Yang J, Li L, Enis D, Ma W, Chen M, Oliver G, Welsh JD et al (2019) Hemostasis stimulates lymphangiogenesis through release and activation of VEGFC. Blood 134:1764–1775 31562136 10.1182/blood.2019001736 PMC6856989

[CR28] Lioux G, Liu X, Temiño S, Oxendine M, Ayala E, Ortega S, Kelly RG, Oliver G, Torres M (2020) A second heart field-derived vasculogenic niche contributes to cardiac lymphatics. Dev Cell 52:350–363.e356 31928974 10.1016/j.devcel.2019.12.006 PMC7374559

[CR29] Liu X, De la Cruz E, Gu X, Balint L, Oxendine-Burns M, Terrones T, Ma W, Kuo HH, Lantz C, Bansal T et al (2020) Lymphoangiocrine signals promote cardiac growth and repair. Nature 588:705–711 33299187 10.1038/s41586-020-2998-x PMC7770123

[CR30] Madisen L, Zwingman TA, Sunkin SM, Oh SW, Zariwala HA, Gu H, Ng LL, Palmiter RD, Hawrylycz MJ, Jones AR et al (2010) A robust and high-throughput Cre reporting and characterization system for the whole mouse brain. Nat Neurosci 13:133–140 20023653 10.1038/nn.2467 PMC2840225

[CR31] Martin M (2011) Cutadapt removes adapter sequences from high-throughput sequencing reads. EMBnet 17:10–12

[CR32] Maruyama K, Miyagawa-Tomita S, Mizukami K, Matsuzaki F, Kurihara H (2019) Isl1-expressing non-venous cell lineage contributes to cardiac lymphatic vessel development. Dev Biol 452:134–143 31112709 10.1016/j.ydbio.2019.05.002

[CR33] Maruyama K, Naemura K, Arima Y, Uchijima Y, Nagao H, Yoshihara K, Singh MK, Uemura A, Matsuzaki F, Yoshida Y et al (2021) Semaphorin3E-PlexinD1 signaling in coronary artery and lymphatic vessel development with clinical implications in myocardial recovery. iScience 24:102305 33870127 10.1016/j.isci.2021.102305 PMC8041864

[CR34] Milasan A, Dallaire F, Mayer G, Martel C (2016) Effects of LDL receptor modulation on lymphatic function. Sci Rep 6:27862 27279328 10.1038/srep27862 PMC4899717

[CR35] Nurmi H, Saharinen P, Zarkada G, Zheng W, Robciuc MR, Alitalo K (2015) VEGF-C is required for intestinal lymphatic vessel maintenance and lipid absorption. EMBO Mol Med 7:1418–1425 26459520 10.15252/emmm.201505731 PMC4644375

[CR36] Paquet-Fifield S, Levy SM, Sato T, Shayan R, Karnezis T, Davydova N, Nowell CJ, Roufail S, Ma GZ, Zhang YF et al (2013) Vascular endothelial growth factor-d modulates caliber and function of initial lymphatics in the dermis. J Invest Dermatol 133:2074–2084 23439394 10.1038/jid.2013.83

[CR37] Pichol-Thievend C, Betterman KL, Liu X, Ma W, Skoczylas R, Lesieur E, Bos FL, Schulte D, Schulte-Merker S, Hogan BM et al (2018) A blood capillary plexus-derived population of progenitor cells contributes to genesis of the dermal lymphatic vasculature during embryonic development. Development 145:dev160184 29773646 10.1242/dev.160184 PMC6001371

[CR38] Ritchie ME, Phipson B, Wu D, Hu Y, Law CW, Shi W, Smyth GK (2015) limma powers differential expression analyses for RNA-sequencing and microarray studies. Nucleic Acids Res 43:e47 25605792 10.1093/nar/gkv007 PMC4402510

[CR39] Trincot CE, Xu W, Zhang H, Kulikauskas MR, Caranasos TG, Jensen BC, Sabine A, Petrova TV, Caron KM (2019) Adrenomedullin induces cardiac lymphangiogenesis after myocardial infarction and regulates cardiac edema via Connexin 43. Circ Res 124:101–113 30582443 10.1161/CIRCRESAHA.118.313835 PMC6318063

[CR40] Tyser RCV, Ibarra-Soria X, McDole K, Arcot Jayaram S, Godwin J, van den Brand TAH, Miranda AMA, Scialdone A, Keller PJ, Marioni JC et al (2021) Characterization of a common progenitor pool of the epicardium and myocardium. Science 371:eabb2986 33414188 10.1126/science.abb2986 PMC7615359

[CR41] Vaahtomeri K, Karaman S, Makinen T, Alitalo K (2017) Lymphangiogenesis guidance by paracrine and pericellular factors. Genes Dev 31:1615–1634 28947496 10.1101/gad.303776.117 PMC5647933

[CR42] Vieira JM, Norman S, Villa Del Campo C, Cahill TJ, Barnette DN, Gunadasa-Rohling M, Johnson LA, Greaves DR, Carr CA, Jackson DG et al (2018) The cardiac lymphatic system stimulates resolution of inflammation following myocardial infarction. J Clin Investig 128:3402–3412 29985167 10.1172/JCI97192 PMC6063482

[CR43] Villa del Campo C, Lioux G, Carmona R, Sierra R, Munoz-Chapuli R, Claveria C, Torres M (2016) Myc overexpression enhances of epicardial contribution to the developing heart and promotes extensive expansion of the cardiomyocyte population. Sci Rep 6:37880 27934852 10.1038/srep37880 PMC5146727

[CR44] Vivien CJ, Pichol-Thievend C, Sim CB, Smith JB, Bower NI, Hogan BM, Hudson JE, Francois M, Porrello ER (2019) Vegfc/d-dependent regulation of the lymphatic vasculature during cardiac regeneration is influenced by injury context. NPJ Regen Med 4:18 31452940 10.1038/s41536-019-0079-2 PMC6706389

[CR45] Wessels A, van den Hoff MJ, Adamo RF, Phelps AL, Lockhart MM, Sauls K, Briggs LE, Norris RA, van Wijk B, Perez-Pomares JM et al (2012) Epicardially derived fibroblasts preferentially contribute to the parietal leaflets of the atrioventricular valves in the murine heart. Dev Biol 366:111–124 22546693 10.1016/j.ydbio.2012.04.020 PMC3358438

[CR46] Wigle JT, Oliver G (1999) Prox1 function is required for the development of the murine lymphatic system. Cell 98:769–778 10499794 10.1016/s0092-8674(00)81511-1

[CR47] Witte MH, Dumont AE, Clauss RH, Rader B, Levine N, Breed ES (1969) Lymph circulation in congestive heart failure: effect of external thoracic duct drainage. Circulation 39:723–733 5785287 10.1161/01.cir.39.6.723

[CR48] Zheng W, Aspelund A, Alitalo K (2014) Lymphangiogenic factors, mechanisms, and applications. J Clin Investig 124:878–887 24590272 10.1172/JCI71603 PMC3934166

