# Supplementary material for: Epicardial VEGFC/D signaling is essential for coronary lymphangiogenesis
Source: EMBO Rep. 2025 Mar 24;26(11):2803–18. doi: 10.1038/s44319-025-00431-7 (PMC12152153; doi:10.1038/s44319-025-00431-7)
Supplement: Supplementary file 2 — Expanded View Figures [file 44319_2025_431_MOESM2_ESM.pdf]

Expanded View Figures

Figure EV1. Characterization of epicardial roles in Lymphangiogenesis.

(**A**) Heart weight to body weight ratios (H/BW) in Control (*Vegf$^{f/f}$*) and epicardial *Vegfc* KO (*WT1^{Cre};Vegf$^{f/f}$*) hearts at postnatal day 20 (P20). (**B**) RNAseq quantification of Cre mRNA expression in dissected epicardial/subepicardial region from *Tbx18^{Cre}*or *WT1^{Cre}* hearts. CPMs: counts per million. (**C**) RNAseq quantification of *Vegfc* mRNA exon 3 expression in dissected epicardial/subepicardial region from Control, *Tbx18^{Cre}*; *Vegfc$^{f/f}$* or *WT1^{Cre}*; *Vegfc$^{f/f}$* hearts (RPKM: Reads Per Kilobase Million). Statistics in (**A**), (**B**), according to the linear modeling in the LIMMA analysis software for RNAseq analysis (see Methods). Benjamini-Hochberg adjusted *P*-values are shown. Graphs show mean ± standard deviation. Each dot represents one biological specimen. $N = 6$ control and 5 *Vegfc−/−* hearts in (**A**) and (**B**). Statistics in (**C**): one-way ANOVA with Sidak's correction for multiple comparisons. Graphs show mean ± standard deviation. Each dot represents one biological specimen. $N = 8$ control, 3 *WT1^{Cre};Vegf$^{f/f}$* and 5 *Tbx18^{Cre}*; *Vegfc$^{f/f}$* hearts. (**D**) Detection of VEGFR3 expression by whole-mount immunofluorescence in wild type E16.5 Hearts. (**E**) Quantification of the mean intensity of VEGFR3 signal in coronary lymphatic vessels of the dorsal and ventral sides of the hearts shown in (**D**). (**F**) Detection of VEGFD expression by whole-mount immunofluorescence in wild type E16.5 Hearts. (**G**) Quantification of the mean intensity of VEGFD signal in coronary lymphatic vessels of the dorsal and ventral sides of the hearts shown in (**F**). (**H**) Detection of Cxcl12 expression by whole-mount immunofluorescence in wild type E16.5 Hearts. (**I**) Quantification of the mean intensity of Cxcl12 signal in coronary lymphatic vessels of the dorsal and ventral sides of the hearts shown in (**H**). Statistics in (**E**, **G**, **I**): Wilcoxon matched-pairs signed rank test with two-tailed *P*-values. Non-significant results are not indicated. Graphs show mean ± standard deviation. Each dot represents one biological specimen. $N = 4$ ventral sides and 5 dorsal sides in (**E**); 5 ventral and dorsal sides in (**G**) and 12 ventral and dorsal sides in (**I**). (**J**) Volcano plot representing the results of RNAseq comparing dorsal and ventral dissected epicardial/subepicardial regions from wild type E16.5 hearts. Genes encoding lymphangiogenic factors and differentially expressed appear highlighted in red. (**K**) Specific results from the RNAseq analysis shown in (**J**) for 22 selected genes encoding lymphangiogenic factors. Statistics in (**J**, **K**), according to the linear modeling in the LIMMA analysis software for RNAseq analysis (see Methods). Benjamini- Hochberg adjusted *P*-values are shown. When not shown, comparsions were not significant (*P*-value > 0.05). Scale bars 100 µm. Each dot represents a biological replicate. $N = 3$ ventral and 3 dorsal sides for all gene expressions represented.

© The Author(s)

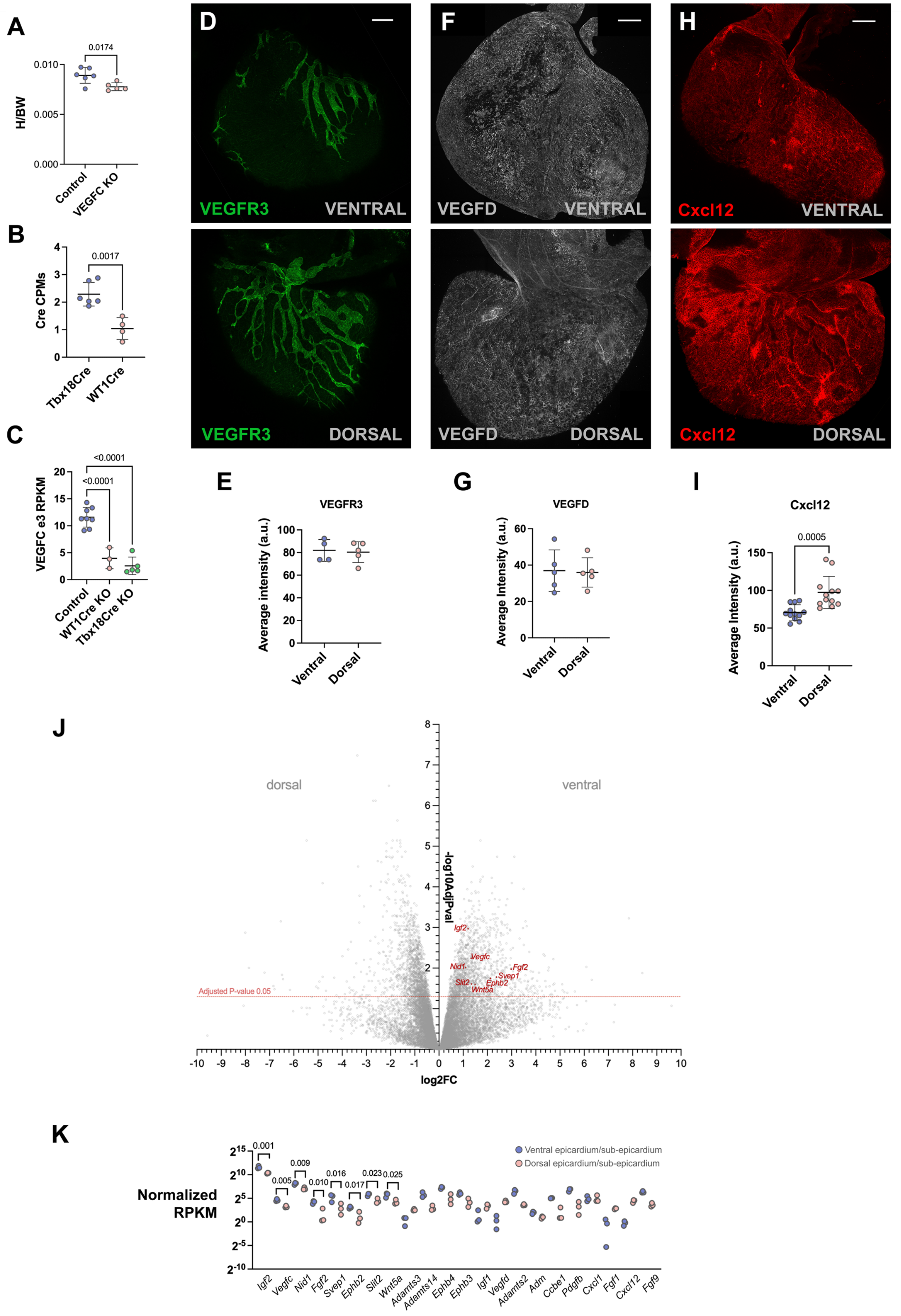
A
0.0174
H/BW
0.010
0.005
0.000
Control
VEGFC KO
B
0.0017
Cre CPMs
Tbx18Cre
WT1Cre
C
<0.0001
<0.0001
VEGFC e3 RPKM
Control
WT1Cre KO
Tbx18Cre KO
D
VEGFR3 VENTRAL
VEGFR3 DORSAL
F
VEGFD VENTRAL
VEGFD DORSAL
H
Cxcl12 VENTRAL
Cxcl12 DORSAL
E
VEGFR3
Average intensity (a.u.)
Ventral
Dorsal
G
VEGFD
Average Intensity (a.u.)
Ventral
Dorsal
I
Cxcl12
0.0005
Average Intensity (a.u.)
Ventral
Dorsal
J
dorsal
ventral
-log10AdjPval
Adjusted P-value 0.05
Igf2
Vegfc
Nid1
Fgf2
Svep1
Slit2
Ephb2
Wnt5a
log2FC
K
Normalized RPKM
Ventral epicardium/sub-epicardium
Dorsal epicardium/sub-epicardium
0.001
0.009
0.005
0.010
0.016
0.017
0.023
0.025
Igf2
Vegfc
Nid1
Fgf2
Svep1
Ephb2
Slit2
Wnt5a
Adamts3
Adamts14
Ephb4
Ephb3
Igf1
Vegfd
Adamts2
Adm
Ccbe1
Pdgfb
Cxcl1
Fgf1
Cxcl12
Fgf9

 © The Author(s)

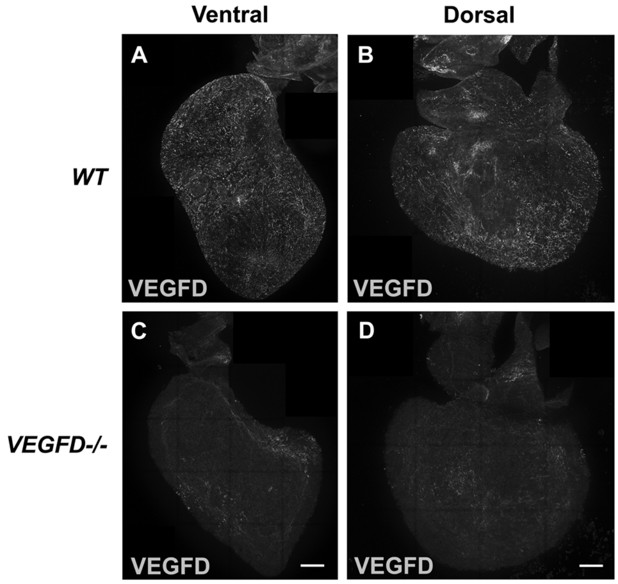

Figure EV2. Characterization of VEGFD epicardial expression.

(**A**, **B**) Detection of VEGFD expression by whole-mount immunofluorescence in wild type E16.5 hearts in ventral (**A**) and dorsal (**B**) views. The specimen in (**A**) is also shown as an example of the VEGFD expression pattern in Fig. 3A. (**C**, **D**) Equivalent detections in *Vegfd* knockout hearts. $N = 5$ Control and 2 VEGFD KO hearts. Scale bars 100 µm.

© The Author(s)

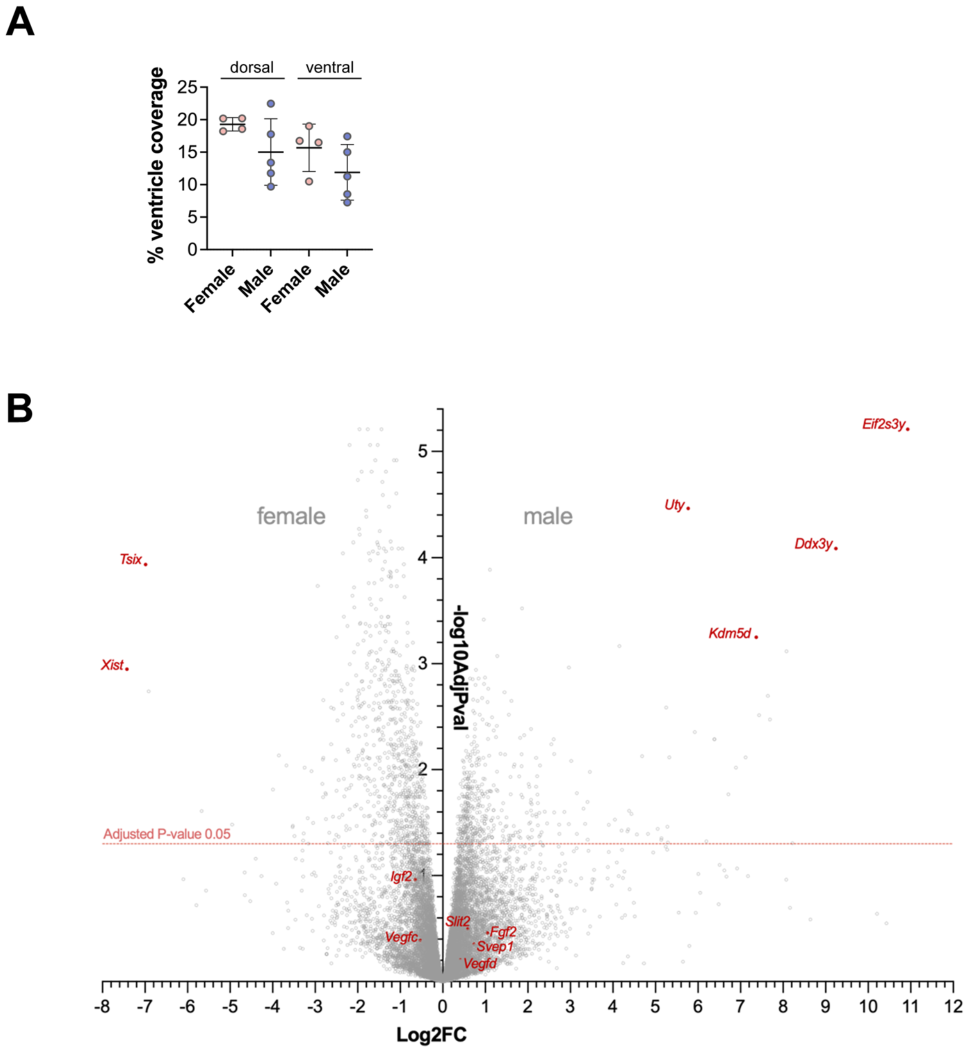

Figure EV3. Analysis of sex-specific features of coronary lymphangiogenesis.

(**A**) Quantification of lymphatic coverage on the ventricles of wild type E16.5 female and male hearts. Statistics: One-way ANOVA with Sidak's multiple comparison test (non-significant). Graphs show mean ± standard deviation. Each dot represents one biological specimen. $N = 4$ female and 5 male samples for both the dorsal and the ventral sides. (**B**) Volcano plot representing the results of RNAseq comparing female to male dissected epicardial/subepicardial regions from wild type E16.5 hearts. Genes encoding for Y-linked and X-chromosome inactivation-specific genes appear in red. Some lymphangiogenic factors showing non-significant differential expression are highlighted in red. Statistics in (**B**), according to the linear modeling in the LIMMA analysis software for RNAseq analysis (see Methods). Benjamini-Hochberg adjusted *P*-values are shown.

 © The Author(s)
